# Liposomal Formulation of an Organogold Complex Enhancing Its Activity as Antimelanoma Agent—In Vitro and In Vivo Studies

**DOI:** 10.3390/pharmaceutics16121566

**Published:** 2024-12-06

**Authors:** Jacinta O. Pinho, Mariana Coelho, Catarina Pimpão, Jahnobi Konwar, Ana Godinho-Santos, Rute M. Noiva, Sophie R. Thomas, Angela Casini, Graça Soveral, Maria Manuela Gaspar

**Affiliations:** 1 Research Institute for Medicines (iMed.ULisboa), Faculty of Pharmacy, Universidade de Lisboa, 1649-003 Lisboa, Portugal; pinho.jacinta@campus.ul.pt (J.O.P.); mariana.coelho@ff.ulisboa.pt (M.C.); pimpaocatarina@gmail.com (C.P.); agsantos@medicina.ulisboa.pt (A.G.-S.); gsoveral@ff.ulisboa.pt (G.S.); 2 Faculty of Pharmacy, Jagiellonian University Medical College, 31-008 Krakow, Poland; jahnobi.konwar@student.uj.edu.pl; 3 CIISA, Interdisciplinary Centre of Research in Animal Health, Faculdade de Medicina Veterinária, Universidade de Lisboa, Av. da Universidade Técnica, 1300-477 Lisboa, Portugal; 4 Department of Chemistry, School of Natural Sciences, Technical University of Munich, 85747 Garching bei München, Germany; sophie.rebecca.thomas@univie.ac.at (S.R.T.); angela.casini@tum.de (A.C.); 5 Faculty of Chemistry, Department of Inorganic Chemistry, University of Vienna, Währinger Straße 42, A-1090 Wien, Austria; 6 IBEB—Institute of Biophysics and Biomedical Engineering, Faculty of Sciences, Universidade de Lisboa, 1749-016 Lisboa, Portugal

**Keywords:** melanoma, gold-based compound, antiproliferative activity, cell cycle arrest, murine melanoma models, lung metastases, therapeutic strategy

## Abstract

**Background/Objectives:** The therapeutic management of melanoma, the most aggressive form of skin cancer, remains challenging. In the search for more effective therapeutic options, metal-based complexes are being investigated for their anticancer properties. Cisplatin was the first clinically approved platinum-based drug and, based on its success, other metals (e.g., gold) are being used to design novel compounds. **Methods:** the antimelanoma potential of a new organometallic cyclometalated Au(III) complex [[Au(C^NOx^N)Cl_2_] (C^NOx^N = 2-(phenyl-(2-pyridinylmethylene)aminoxy acetic acid))] (ST004) was evaluated in vitro and in vivo. Furthermore, the gold-based complex was incorporated in liposomes to overcome solubility and stability problems, to promote accumulation at melanoma sites and to maximize the therapeutic effect while controlling its reactivity. The antiproliferative activity of ST004 formulations was assessed in murine (B16F10) and human (A375 and MNT-1) melanoma cell lines after 24 and 48 h incubation periods. The proof-of-concept of the antimelanoma properties of ST004 formulations was carried out in subcutaneous and metastatic murine melanoma models. **Results:** the developed liposomal formulations showed a low mean size (around 100 nm), high homogeneity (with a low polydispersity index) and high incorporation efficiency (51 ± 15%). ST004 formulations exhibited antiproliferative activity with EC_50_ values in the μmolar range being cell-line- and incubation-period-dependent. On the opposite side, the benchmark antimelanoma compound, dacarbazine (DTIC), presented an EC_50_ > 100 μM. Cell cycle analysis revealed an arrest in G0/G1 phase for Free-ST004 in all cell lines. In turn, LIP-ST004 led to a G0/G1 halt in B16F10, and to an arrest in S phase in A375 and MNT-1 cells. Preliminary mechanistic studies in human red blood cells suggest that gold-based inhibition of glycerol permeation acts through aquaglyceroporin 3 (AQP3). In a metastatic murine melanoma, a significant reduction in lung metastases in animals receiving LIP-ST004, compared to free gold complex and DTIC, was observed. **Conclusion:** This study highlights the antimelanoma potential of a new gold-based complex. Additional studies, namely in vivo biodistribution profile and therapeutic validation of this organogold complex in other melanoma models, are expected to be performed in further investigations.

## 1. Introduction

Melanoma, a highly aggressive form of skin malignancy, accounts for the 18th most common form of cancer worldwide [1]. In early-stage disease, surgical excision is the primary therapeutic option. In turn, advanced melanoma is often untreatable, being associated with an extremely aggressive profile and low survival rates [2,3]. Other therapeutic strategies are radiotherapy [4], chemotherapy [5], immunotherapy [6,7], targeted therapy [6,8,9] and more recently, tumor-infiltrating lymphocyte therapy [10]. Chemotherapy is the most employed anticancer strategy, although some limitations are associated with chemotherapeutic agents, namely low specificity, toxic side effects and development of resistance [2]. Nevertheless, for advanced melanoma, DTIC constitutes the only chemotherapeutic drug approved by U.S. Food and Drug Administration (FDA) [11]. To tackle these challenges, metal coordination is receiving special emphasis since it offers new therapeutic opportunities that are inaccessible to conventional organic or biological compounds [12].

Although metal-based compounds have successfully impacted medicine in different areas, in anticancer therapy, only the platinum(II)-based drugs cisplatin, carboplatin and oxaliplatin are clinically approved and used in about 50% of all cancer chemotherapeutic regimens [12,13,14]. Nevertheless, the emergence of platinum resistance in cancer is encouraging researchers to design novel and improved molecules [2,13,15,16,17]. In melanoma, different metals are being investigated for the development of drug candidates, including copper [18,19,20], gold [21,22,23], silver [21], zinc [19], vanadium [24], and ruthenium [25,26], among others.

**Figure 1:**
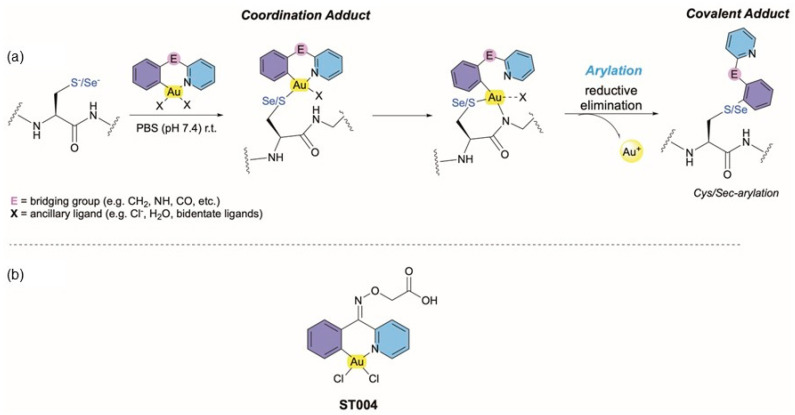
(**a**) Proposed scheme of the reaction of C^N-cyclometalated Au(III) compounds with thiols and selenols of proteins. Different bridging groups (E) modulate the propensity of the compound toward cysteine arylation. Moreover, the reactivity of the compound can also be modulated by the ancillary ligands. The binding to selenol/thiolate groups of proteins proceeds via two steps: (i) the formation of a coordination adduct in which the gold(III) center binds directly to the S-/Se-nucleophiles, and (ii) C-S/Se cross-coupling reaction via reductive elimination and liberation of Au(0/I). (**b**) Structure of the tested organogold compound, [[Au(CNOxN)Cl2] (CNOxN = 2-(phenyl-(2-pyridinylmethylene)aminoxy acetic acid))], ST004.

Gold-based complexes are highlighted due to their versatility in terms of electronic structures and variable redox states that grant them diversified biological activities and high therapeutic potential. Since the 2000s, the use of gold in medicinal chemistry has exponentially increased, and different families of Au(I) and Au(III) complexes have been investigated for their new mechanisms of action and possible pharmacological targets [27,28,29]. Auranofin, an alkylphosphine Au(I) drug, was approved by the FDA in 1985 for the treatment of rheumatoid arthritis [27]. Currently, there are ongoing clinical trials with auranofin to repurpose it for application in other diseases, including cancer [27,30]. In this context, gold-based compounds as anticancer agents have been widely investigated by various groups, including coordination and organometallic derivatives [31,32,33]. Recently, it has been shown that organometallic Au(III) complexes may exert their anticancer activity by covalent targeting of proteins beyond the pure coordinative binding mode [34,35,36,37,38], and as such emerge as new chemical tools for bioorthogonal transformations in vitro and in vivo, as well as novel drug leads. For example, cyclometalated gold(III) compounds featuring bidentate C^N ligands and different ancillary ligands, can template the formation of covalent aryl-peptide adducts via C–S or C–Se cross-coupling at cysteine/selenocysteine residues (Figure 1a) [37,39,40]. Amongst the possible targets, the covalent inhibition of the cancer relevant selenoenzyme thioredoxin reductase 1 (TXNRD1) [40] and of membrane transporters of water and glycerol (aquaglyceroporins, AQPs) [41] by cyclometalated gold(III) compounds have been recently reported. In general, several studies by our groups have demonstrated that gold-based complexes are potent and selective inhibitors of aquaporin-3 (AQP3), a membrane transporter of water and glycerol that is overexpressed in melanoma [42,43,44,45]. The ability of gold-based complexes to modulate AQP3 activity has made them promising molecules against cancer and particularly for melanoma management [46].

The main factors hindering the clinical translation of gold-based compounds are their limited solubility and chemical instability in physiological milieu. These challenges may be overcome on one hand using the aforementioned organometallic scaffolds, which, featuring a direct C–Au bond, can stabilize even the highest possible Au(III) oxidation state. On the other hand, further stabilization of the gold compound’s reactivity can be achieved through the incorporation into drug delivery systems. Combining these strategies, the compound’s pharmacological properties are retained, and its in vivo application is facilitated [28,47]. Liposomes, in particular, are the most successful and versatile lipid-based nanosystems, with numerous products approved for clinical use or undergoing clinical trials [48,49,50]. These lipid-based nanosystems provide protection against premature degradation and prevent undesirable effects on healthy tissues. Moreover, the surface can be modified with polyethylene glycol (PEG) to increase blood circulating times and/or functionalized with specific ligands to promote active targeting of tissues/cells [2,18,51,52]. Clinical trials of metal-based nanomedicines are limited to platinum drugs loaded in liposomes (Lipoplatin, Aroplatin, SPI-077, and LiPlaCis) [53] and polymeric micelles (NC-6004) [54]. Despite the benefits of using delivery systems for metal-based compounds, research on nanomedicines of gold-based compounds is scarce [28]. The aim of the present work was to assess the in vitro and in vivo antimelanoma potential of the Au(III) cyclometalated complex [[Au(C^NOx^N)Cl_2_] (C^NOx^N = 2-(phenyl-(2-pyridinylmethylene)aminoxy acetic acid))] [41], hereby designated as ST004 (Figure 1), when in free form and after association with long blood-circulating liposomes (LIP-ST004). The compound has been previously shown to react by a two-step mechanism involving reversible coordination of the cyclometalated gold(III) scaffold to thiolates of target cysteine residues, followed by reductive elimination and irreversible covalent cross-coupling reaction of the ligand to these nucleophiles. The presence of the free carboxylic acid group in the backbone enables its conjugation to additional functionalities (e.g., fluorophores [40] or targeting moieties) for further optimization of the compound’s bioactivity. The stability of ST004 was previously assessed by ^1^H NMR spectroscopy in a DMSO-d_6_/D_2_O (9:1) solvent mixture at room temperature [40]. In the presence of GSH, the compound could template the arylation of the GSH thiols, although this side-reactivity did not prevent it from potent and selective arylation of specific proteins in cancer cells [40]. In any case, one of the advantages of the liposomal formulation is that it can protect the metallodrug from off-target reactivity, particularly extracellularly.

The inhibitory potency of ST004 on AQP3 was evaluated in human red blood cells (RBCs) that endogenously express AQP3. The upregulation of AQP3 has been reported in melanoma [55]. Therefore, cytotoxicity and cell cycle assays of ST004 liposome formulations were performed in murine (B16F10) and human (A375 and MNT-1) melanoma cell lines. The gene expression of AQP3 has previously been assessed [46,56], with MNT-1 cells presenting higher transcript levels than A375 cells [46]. The in vivo proof-of-concept was conducted in B16F10 murine models of subcutaneous and metastatic melanoma.

## 2. Materials and Methods

### 2.1. Reagents

The gold compound, ST004, was provided by Angela Casini, from the Technical University of Munich and synthesized according to a previously published procedure [40]. Dimethyl sulfoxide (DMSO) and 3-(4,5-dimethylthiazol-2-yl)–2,5-diphenyltetrazolium bromide (MTT) were acquired from Sigma-Aldrich (St Louis, MO, USA). The pure phospholipids, dimyristoyl phosphatidyl choline (DMPC; MW = 678), dioleoyl phosphatidyl ethanolamine (DOPE; MW = 744) and distearoyl phosphatidyl ethanolamine covalently linked to poly(ethylene glycol) 2000 (DSPE-PEG; MW = 2790) were purchased from Avanti Polar Lipids (Alabaster, AL, USA). Deionized water (Milli-Q system; Millipore, Tokyo, Japan) was used in all experiments. Nuclepore Track-Etched membranes were acquired from Whatman Ltd. (New York, NY, USA). Culture media and antibiotics were obtained from Invitrogen (Thermo Fisher Scientific, Waltham, MA, USA). Propidium iodide (PI) was obtained from Thermo Fisher Scientific (Eugene, OR, USA) and RNase A was purchased from Sigma-Aldrich (Darmstadt, Germany). Reagents for cell proliferation assays were purchased from Promega (Madison, WI, USA). All the remaining chemicals used were of analytical grade.

### 2.2. Cell Lines Culture Conditions

Human melanoma A375 (ATCC^®^ CRL-1619™) and MNT-1 (ATCC^®^ CRL-3450™) and murine melanoma B16F10 (ATCC^®^ CRL-6475™) cell lines were maintained in Dulbecco’s modified Eagle’s medium (DMEM) with high-glucose (4500 mg/L), supplemented with 10% fetal bovine serum (FBS) and 100 IU/mL of penicillin and 100 μg/mL streptomycin (Gibco, Thermo Fisher Scientific, Waltham, MA, USA). Cells were maintained in 75 cm^2^ culture flasks at 37 °C in a humidified air incubator, at 5% CO_2_.

### 2.3. Red Blood Cells Sampling and Preparation

Venous blood samples were collected from anonymous human donors in citrate anticoagulant (2.7% citric acid, 4.5% trisodium citrate, and 2% glucose) to prevent coagulation. The blood was centrifuged at 750× *g* for 10 min at room temperature (RT) to isolate red blood cells (RBCs). After washing three times with PBS, RBCs were diluted to a 0.5% suspension and kept on ice to be immediately used in the experiments.

### 2.4. Animals

Male C57BL/6 (8–10-week-old) mice were purchased from Charles River (Barcelona, Spain). Animals were kept in ventilated cages under standard hygiene conditions, on a 12 h light/12 h dark cycle, at 20–24 °C and 50–65% humidity. Mice had ad libitum intake of sterilized diet and acidified water. All in vivo experimental protocols were conducted according to the animal welfare organ of the Faculty of Pharmacy, Universidade de Lisboa, approved by the competent national authority Direção-Geral de Alimentação e Veterinária (DGAV) and in accordance with the EU Directive (2010/63/UE) and Portuguese laws (DR 113/2013, 2880/2015, 260/2016 and 1/2019) for the use and care of animals in research.

### 2.5. Preparation and Physicochemical Characterization of ST004 Liposomes

Liposomes were prepared by the dehydration–rehydration method. The selected phospholipids (30 μmol/mL) and ST004 (1000 μg/mL) were dissolved in chloroform, evaporated (Buchi R-200 rotary evaporator, Buchi, Flawil, Switzerland) in a round-bottomed flask to achieve a lipid film. The so formed lipid film was then dispersed with deionized water and the suspension was frozen and lyophilized in a freeze dryer (Edwards Micro Modulyo, Edwards, CO, USA) overnight. The lyophilized powder was rehydrated in HEPES buffer pH 7.4 (10 mM HEPES, 140 mM NaCl) in two steps, at a temperature higher than the phase transition temperature (Tc) of the phospholipid, DMPC, the main component of the lipid composition. The so-formed liposomal suspension was then filtered under nitrogen pressure (10–500 lb/in2), through Nuclepore Track-Etched polycarbonate membranes (Whatman Ltd., New York, NY, USA) of appropriate pore size (0.6, 04, 0.2, 0.1 μm), with 2–3 cycles of extrusion for each membrane to achieve an average mean size of 100 nm, using an extruder device (Lipex Biomembranes Inc., Vancouver, Canada). The separation of non-incorporated ST004 was performed by gel filtration (Econo-Pac^®^ 10DG; Bio-Rad Laboratories, Hercules, CA, USA), followed by ultracentrifugation at 250,000 g, for 120 min, at 15 °C in a Beckman LM-80 ultracentrifuge (Beckman Instruments, Inc., Fullerton, CA, USA). The pellet was finally suspended in HEPES buffer pH 7.4. Liposomes were characterized in terms of incorporation parameters, mean size and zeta potential. Loading capacity was defined as the final ST004 to lipid ratio (ST004/lipid)f and the incorporation efficiency (I.E.), in percentage, was determined using Equation (1):

(1) $$I.E.\left(\%\right)=\frac{\left(\frac{\mathrm{ST}004}{\mathrm{lipid}}\right)f}{\left(\frac{\mathrm{ST}004}{\mathrm{lipid}}\right)i}\times 100$$

ST004 was quantified spectrophotometrically at λ = 283 nm after disruption of liposomes with ethanol. Linearity of calibration curves of ST004 was ensured from 5 to 30 μM. Lipid content was determined according to Rouser et al. with some modifications [57]. The full description of the methodology can be found in [58]. Liposome mean size and polydispersity index (PdI) were determined by dynamic light scattering (Zetasizer Nano Series, Nano-S Malvern Instruments, Malvern, UK), and zeta potential was measured by laser Doppler electrophoresis (Zetasizer Nano Series, Nano-Z Malvern Instruments, Malvern, UK).

### 2.6. Cell Viability Assay

For the in vitro assessment of ST004 formulations antiproliferative activity, human (A375 and MNT-1) and murine (B16F10) melanoma cell lines were seeded in 96-well plates at a density of 5 × 10^4^ cells/mL, 200 μL/well. After 24 h at 37 °C, 5% CO_2_, cells were incubated with ST004 in free and liposomal forms at concentrations ranging from 10–100 μM. Negative controls were cells in the presence of complete medium and the positive control corresponded to cells incubated with dacarbazine (DTIC) at concentrations ranging from 20 to 100 μM. Unloaded liposomes were also tested at the same lipid concentrations corresponding to ST004-loaded liposomes. Following 24 h and 48 h incubation, medium was removed, and wells were washed with PBS (200 μL/well). Then, 50 μL of the MTT solution (0.5 mg/mL of non-FBS medium) were added to each well and incubated at 37 °C, for 1–2 h. The resulting formazan crystals were solubilized with 100 μL of DMSO and absorbance was read at 570 nm. Cell viability analysis was performed using GraphPad Prism^®^8.0.1 for Windows (GraphPad Software, San Diego, CA, USA, “www.graphpad.com” accessed on 23 July 2024) To determine the EC_50_ values, data were plotted and fit to a standard inhibition log concentration-response curve.

### 2.7. Cell Cycle Analysis

Cell cycle distribution assay was performed using a propidium iodide (PI) staining protocol, followed by flow cytometry analysis [51]. Briefly, melanoma cell lines were seeded in 6-well plates at a density of 5 × 10^4^ cells/mL, 3 mL/well. After 24 h, ST004 formulations were incubated at 50, 30 and 40 μM in B16F10, A375 and MNT-1, respectively. The positive control DTIC was tested at 70 μM in all cell lines [51]. Negative control were cells in the presence of complete culture medium. Following 24 h of incubation, cells were detached and collected. After a centrifugation step at 800 *g* for 5 min at 4 °C, cells were suspended in cold PBS (500 μL) and an equal volume of 80% ice-cold ethanol (−20 °C) was added drop by drop, with gentle agitation. Samples were kept at 4 °C until analysis. For data acquisition, cells were centrifuged at 850 *g* for 5 min, at 4 °C. Cell pellets were suspended in 300 μL of cold PBS with PI and RNase A at a final concentration of 25 and 50 μg/mL, respectively, and incubated at 37 °C for 30 min. Acquisition of 40,000 events per sample was performed on a full-spectrum Cytek^®^ Aurora cytometer (Cytek Biosciences, Inc., Fremont, CA, USA). Cell cycle histograms were automatically generated for each sample using the MultiCycle AV in FCS Express^TM^ 7 Software, version 7.20.0020 (DeNovo Software, Pasadena, CA, USA).

### 2.8. Inhibition of AQP3 Glycerol Permeability

To evaluate the inhibitory potency of ST004 on AQP3, we performed glycerol permeability assays using human RBCs by light scattering stopped-flow spectroscopy [44] using a HI-TECH Scientific PQ/SF-53 stopped-flow apparatus interfaced with a microcomputer. After challenging cell suspensions with an equal volume of a glycerol hyperosmotic solution (200 mM glycerol in PBS pH 7.4) at 23 °C, creating an inwardly directed glycerol gradient, the time course of volume changes was measured following, at 400 nm, the 90° scattered light intensity. After the first fast cell shrinkage, due to water outflow, glycerol influx in response to its chemical gradient was followed by water influx with subsequent cell reswelling. For each experimental condition, 5 to 7 replicates were carried out. Baselines were acquired using the respective incubation buffers as isotonic shock solutions.

Glycerol permeability (P_gly_) was calculated according to Equation (2),

P_gly_ = k (V_0_/A) (2)

where V_0_/A is the initial cell volume to area ratio, and k is the single exponential time constant fitted to the light scattering signal of glycerol influx in erythrocytes [44].

To assess the inhibitory potency of ST004 on AQP3 activity, cells were incubated with different concentrations of the compound (up to 100 µM) for 30 min at RT before permeability experiments. The inhibitor concentration that corresponds to 50% inhibition (IC_50_) was calculated by nonlinear regression of dose–response curves (GraphPad Prism software) according to Equation (3),

y = 100/(1 + 10((LogIC_50_ − x) × HillSlope)) (3)

where HillSlope describes the steepness of the family of curves.

### 2.9. Therapeutic Evaluation of ST004 Formulations in Murine Models of Melanoma

Subcutaneous (s.c.) and metastatic melanoma tumors were induced with 1.3 × 10^5^ and 4 × 10^6^ B16F10 murine melanoma cancer cells, respectively. Cells were suspended in 100 μL PBS and injected subcutaneously in the right flank of mice for the s.c. model [18,51,52] or intravenously in the tail vein for the metastatic melanoma [51]. Animals were monitored for pain or distress and weighed daily. Animals were sacrificed when they met the ethical euthanasia criteria for overall health condition (loss of weight > to 20%, showing signs of morbidity or tumor volumes > 2000 mm^3^ [59,60]).

In the s.c. model, tumors became palpable on day 12 after induction and treatment protocol was initiated. Experimental groups were randomly organized (n = 5) and mice received tested formulations by intravenous (i.v.) administration, one per day, for five consecutive days: Negative control group received PBS (Control); Free-ST004 and LIP-ST004 groups received formulations at 3.5 mg/kg of body weight. Tumor dimensions were regularly assessed using a digital caliper. Two days after the last treatment, mice were sacrificed, and organs of interest were collected for further analysis. Tumor volumes were calculated according to Equation (4),

(4) $${V(\mathrm{mm}}^{3})=\frac{\left({L\times W}^{2}\right)}{2}$$

where L and W represent the longest and shortest axis of the tumor, respectively. Relative tumor volumes (RTV) were determined for each animal, as the ratio between volumes at the indicated day and volumes at the beginning of treatment.

In the metastatic melanoma model, seven days post-induction, mice were randomly divided in groups of 6 and received the formulations under study by i.v. route for five consecutive days: Negative control group received PBS (Control); positive control group received DTIC at 10 mg/kg of body weight; Free-ST004 and LIP-ST004 were injected at 3.5 mg/kg body weight. At the end of the experimental protocol, lungs were macroscopically analyzed, and a score based on the number of metastases was established: 1 = 0–5 metastases; 2 = 6–20 metastases; 3 = 21–50 metastases; 4 = 51–100 metastases. For histopathological analysis, samples of lungs were preserved in 10% buffered formalin for bread-slice total routine processing and paraffin embedding. Three micrometer sections were stained with Hematoxylin and Eosin (H&E) for morphological analysis under optical microscopy. A board-certified veterinary pathologist that was blinded to the treatment group evaluated the slides for metastasis identification. Sample examination and image capture were performed on an Olympus BX51 microscope equipped with a DP21 camera (Olympus).

In both murine models, the safety of tested formulations was evaluated through tissue index and serum levels of aspartate transaminase (AST) and alanine transaminase (ALT). Organs of interest (liver, spleen, kidneys, and lungs) were collected and weighed for tissue index determination according to Equation (5).

(5) $$\mathrm{Tissue}\;\mathrm{index}=\sqrt{\frac{\mathrm{organ}\;\mathrm{weight}}{\mathrm{animal}\;\mathrm{weight}}}\times 100$$

For hepatic enzymes assay, serum was isolated from the blood, and AST and ALT levels were analyzed using a commercially available kit (Spinreact, Spain).

### 2.10. Statistics

Two-way ANOVA with Tukey’s test and unpaired *t*-test with Welch’s correction were performed using GraphPad Prism^®^8 (GraphPad Software, San Diego, CA, USA). Statistical significance was considered at *p* < 0.05.

## 3. Results

### 3.1. Physicochemical Characterization of ST004 Liposomes

Despite their promise as anticancer agents, gold-based compounds display poor bioavailability and limited chemical stability in physiological milieu, which hamper their successful clinical translation [22,28]. To overcome these challenges, lipid-based nanosystems, namely liposomes, are advantageous tools for the effective protection of associated and delivery of compounds. Liposomes as drug delivery systems were able to revolutionize the pharmaceutical and medical fields, being the first that have successfully reached the clinic [49]. Metal-based complexes have been successfully loaded in liposomes by the team [18,58], particularly those presenting pH sensitive properties [61], composed by DMPC:DOPE:CHEMS:DSPE-PEG. Regarding the gold-based complex ST004, preliminary studies demonstrated that this lipid composition was not adequate in terms of stability of the compound [62], and so the selected one did not include CHEMS. Aiming to maximize the loading of ST004 in liposomes two initial concentrations of ST004 were tested: 400 and 1000 μg/mL. The obtained results are depicted in Table 1.

Overall, ST004 liposomes presented a low mean size (ca. 100 nm) and high homogeneity (PdI < 0.1), physicochemical properties that are achieved based on the method used for their preparation [63]. The presence of the polymer DSPE-PEG conferred a surface charge close to neutrality, as confirmed by the zeta potential values of −4 and −5 mV. The loading capacity was positively correlated with the initial ST004 concentration. The higher initial ST004 concentration of 1000 μg/mL led to an increased loading capacity (15 ± 5 μg/μmol of lipid) compared to liposomes prepared with an initial concentration of 400 μg/mL (5 ± 1 μg/μmol of lipid). Therefore, liposomes prepared with the initial ST004 concentration of 1000 μg/mL were selected for further in vitro and in vivo testing.

### 3.2. ST004 Formulations Display In Vitro Antiproliferative Activity Towards Melanoma Cell Lines

**Figure 2:**
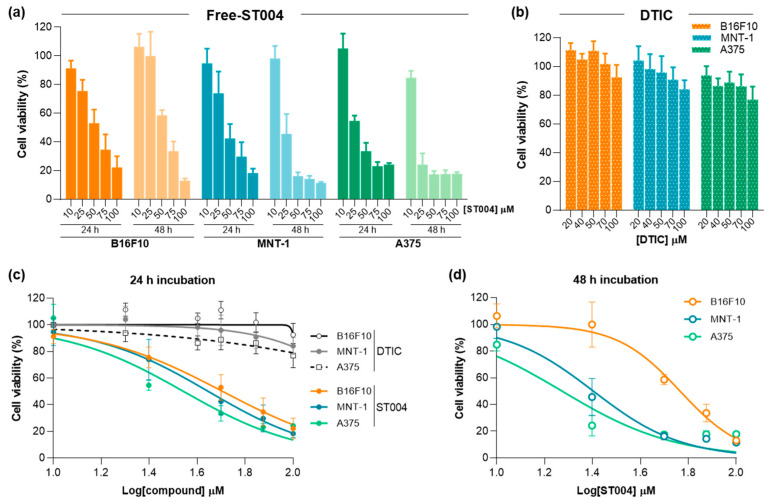
Antiproliferative activity of Free-ST004 and DTIC against melanoma cell lines B16F10, MNT-1 and A375. (**a**) Cell viability after 24 and 48 h incubation with ST004 in the free form at concentrations ranging from 10 to 100 μM. (**b**) Cell viability after 24 h incubation with DTIC at concentrations ranging from 20 to 100 μM. (**c**,**d**) Concentration–response curves of melanoma cell lines after 24 h (**c**) and 48 h (**d**) incubation with tested formulations. Results are expressed as mean ± SD (n = 2–3). DTIC: dacarbazine.

**Figure 3:**
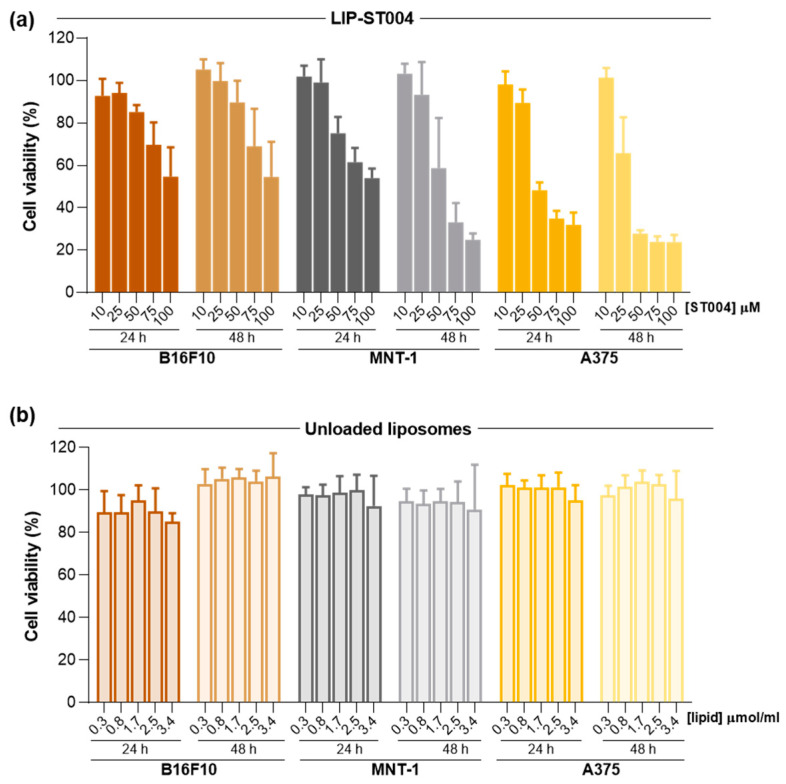
Antiproliferative activity of LIP-ST004 against melanoma cell lines B16F10, MNT-1 and A375. Data correspond to cell viability after 24 and 48 h incubation with (**a**) LIP-ST004 at concentrations ranging from 10 to 100 μM; (**b**) unloaded liposomes were tested at the same lipid concentrations as ST004-loaded liposomes. Results are expressed as mean ± SD (n = 2). LIP-ST004: DMPC:DOPE:DSPE-PEG (50:45:5).

In the present work, the anticancer properties of this new gold-based complex were investigated in both free and liposomal forms. Additionally, DTIC was tested as benchmark drug to treat melanoma [64]. Results of cell viability after incubation with Free-ST004 and DTIC are depicted in Figure 2 and data from LIP-ST004 are depicted in Figure 3, with the corresponding unloaded lipid composition. The EC_50_ values of tested formulations are summarized in Table 2.

ST004 in both free and liposomal forms demonstrated a concentration- and time-dependent antiproliferative activity in all melanoma cell lines, with an overall decrease in the EC_50_ values at 48 h when compared to the 24 h incubation period (Table 2). In all tested conditions, Free-ST004 displayed higher cytotoxicity compared to LIP-ST004. The lowest EC_50_ was obtained for Free-ST004 in A375 cells at 48 h incubation (17 ± 1 μM) and the highest value was observed for LIP-ST004 in MNT-1 cell line after 24 h incubation (95 ± 3 μM). Recent work with four different Au(III) complexes revealed cell viabilities of around 70–100% and 80–90% for MNT-1 and A375, respectively, at the maximum tested concentration of 10 μM, after 24 h incubation [46]. These results are in line with previous reports on different cyclometallated Au(III) [40,65], showing a concentration- and time-dependent effect of ST004 on melanoma cell lines.

The superior antiproliferative activity of Free-ST004 was expected, since, in free form, ST004 is readily available to act on intracellular and extracellular targets, opposed to compound in liposomal form, and may display different cell uptake mechanisms. This has also been observed in other research studies where liposomal nanoformulations displayed decreased cytotoxic activity compared to free compounds [51,66]. Of note, unloaded liposomes tested at the same lipid concentrations as the corresponding ST004-loaded liposomes did not affect cell viability (Figure 3b). This confirmed that the observed antiproliferative activity of LIP-ST004 was solely due to the gold-based complex. In turn, the positive control DTIC revealed a weak antiproliferative activity, with an EC_50_ > 100 μM for all tested cell lines, after 24 h incubation. This is in agreement with previous literature reports using melanoma cell lines [52,67,68]. This effect might be explained by the fact that DTIC is a triazene prodrug, that acts as an alkylating agent after activation in the liver [69].

### 3.3. ST004 Formulations Exert Cell Cycle Alterations in Melanoma Cells

**Figure 4:**
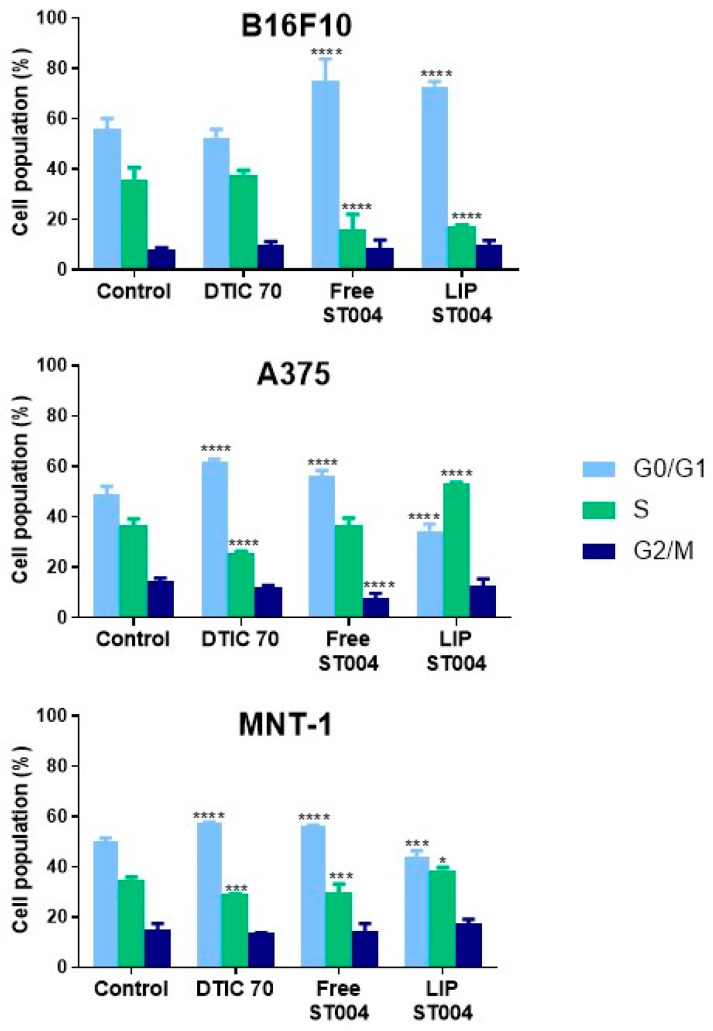
Quantitative analysis of gated B16F10, A375 and MNT-1 melanoma cell lines cells in the G0/G1, S, and G2/M cell cycle phases in the absence (Control) or presence of DTIC at 70 μM (DTIC 70) or ST004 in free (Free-ST004) and liposomal (LIP-ST004) forms at 50, 30 and 40 μM for B16F10, A375 and MNT-1, respectively. LIP-ST004: DMPC:DOPE:DSPE-PEG(50:45:5). Results are expressed as mean ± SD (n = 3). Statistical analysis was performed using two-way ANOVA with Tukey’s test. * *p* < 0.05, *** *p* < 0.001 and **** *p* < 0.0001 vs. Control.

It is well established that cell cycle regulation is a key process in tumorigenesis and cancer response to therapy. A major goal of chemotherapy is to prevent tumor progression, which may be achieved by interfering with one or more phases of the cell cycle [70]. Here, the effect of tested formulations on cell cycle progression was investigated. For that, melanoma cell lines were incubated with Free-ST004 at concentrations close to the 24 h EC_50_ values: 50, 30 and 40 μM for B16F10, A375 and MNT-1 cells, respectively. LIP-ST004 was tested at the same concentrations as the free gold-based complex to allow the comparison of obtained results (Figure 4). The concentration of the positive control, DTIC, was 70 μM for all cell lines [51].

Incubation with tested compounds for 24 h led to alterations in the cell cycle of melanoma cells when compared to controls (Figure 4 and Supplementary Information, Figures S1 and S2). The positive control, DTIC, is an alkylating agent that affects cell independently of the phase [71]. For human melanoma cell lines under study, DTIC, the positive control, at 70 μM caused an arrest at G0/G1 phase, with a concomitant decrease in S phase population. While a similar effect has been previously reported in A375 cells [52,72], other researchers have noted different cell cycle responses for DTIC in these cell lines but using lower or much higher concentrations. For instance, DTIC incubation with A375 (ca. 30 μM) and MNT-1 (ca. 631 μM) resulted in no effects or increased S phase cell population, respectively [73]. In another work using B16F10 cells, a concentration-dependent effect was observed for DTIC. At ca. 137 μM and 412 μM, this drug led to a G1 and a G2 phase arrest, respectively [74].

Incubation with Free-ST004 resulted in a significant increase in G0/G1 population in tested cell lines, being more evident in B16F10. Previous work with the Au(III) complex Auphen, an inhibitor of aquaporin (AQP)3- and AQP10-facilitated glycerol permeation, has been reported [41,75]. Cell cycle analysis in AQP3-expressing PC12 cells revealed accumulation in S-G2/M phases after incubation with Auphen [75]. Moreover, while different cyclometalated Au(III) complexes have induced G2/M arrest in colorectal cancer cells [76], other types of Au(III) cyclometalated compounds induced a G1 halt in breast cancer MDA-MB-231 cell line [77].

In turn, LIP-ST004 activity was cell-line-dependent. While in B16F10 cells, a G0/G1 phase halt occurred, in A375 and MNT-1, a significant increase in the cell population at S phase was observed (Figure 4 and Supplementary Information, Figure S2). In the literature, distinct effects of liposomal formulations depending on the cell line have been reported. In a study with liposomes loading SN-38, the active metabolite of irinotecan, HeLa and Caco-2 cells displayed a sub-G1 and S phase arrest, respectively [78].

Furthermore, the association of compounds to liposomes has been described to modify their activity on cell cycle. For instance, while liposomal C6 ceramide did not affect cell cycle progression [79], the free form was shown to block cells at G1 phase [80]. In addition, in A549 cells, liposomal gefitinib induced a significantly more pronounced arrest in G0/G1 phase, compared to the free drug [81]. Further, when associated with positively charged liposomes, the drug 6-mercaptopurine caused a sub-G0/G1 arrest in HepG2 cells. In turn, the free drug stopped cell cycle progression at G2/M phase [82]. Another example is the liposomal form of hydrogenated anacardic acid that led to a cell cycle halt at sub-G1 and G2/M phases in cancer stem cells, whereas the free form only increased sub-G1 phase [83].

### 3.4. ST004 Inhibits AQP3 Activity

**Figure 5:**
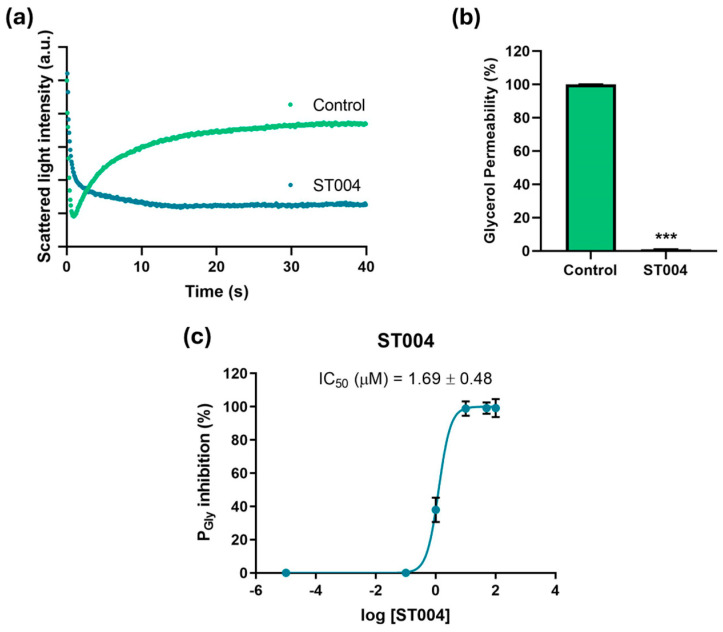
Effect of ST004 on AQP3 activity in human red blood cells (RBCs). (**a**) Representative stopped-flow signal showing changes in scattering light intensity when cells are confronted with a hyperosmotic glycerol solution. After a first shrinkage due to water efflux, cells reswell due to glycerol entrance via aquaporin 3 (AQP3) (control). Cell treatment with ST004 impairs glycerol influx. (**b**) Glycerol permeability of RBCs incubated with ST004 (100 µM for 30 min). (**c**) Dose-response curves of AQP3 glycerol permeability inhibition by ST004 (0–100 µM). Data are shown as means ± SD of three independent experiments. *** *p* < 0.001 vs control.

To gain preliminary insights into the mechanism of action of ST004, we investigated its potential inhibitory effect on AQP3 by measuring the glycerol permeability on a simple model consisting of human RBCs and using stopped-flow light scattering. Cells were challenged with a hyperosmotic glycerol solution inducing water efflux and cell shrinkage, followed by cell reswelling due to glycerol entrance via AQP3 (Figure 5a). Glycerol permeability (Pgly) was calculated from the rate of cell reswelling in before and after cell treatment with the organogold compound ST004 (Figure 5b).

Cells treated with ST004 exhibited an impaired glycerol permeability, revealing the strong inhibitory effect of ST004 on AQP3-mediated glycerol transport. Moreover, we assessed glycerol permeation of RBCs treated with different concentrations of ST004 (0 to 100 µM) revealing that ST004 is a potent inhibitor of AQP3 activity, with a low IC_50_ (1.69 ± 0.48 µM) (Figure 5c). Considering that AQP3 is overexpressed in melanoma, inhibiting AQP3 function may explain, at least in part, the potential of ST004 as an anticancer drug for melanoma.

### 3.5. Therapeutic Evaluation of ST004 Formulations in Subcutaneous and Metastatic Murine Melanoma Models

**Figure 6:**
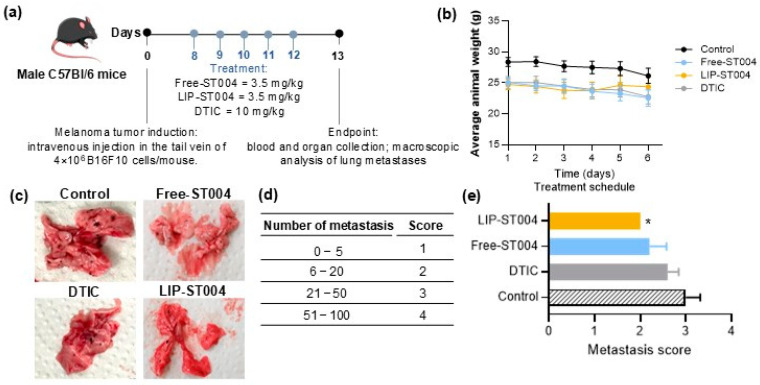
Therapeutic effect of tested formulations in a metastatic murine melanoma model. Tumor induction was performed by an i.v. inoculation of 4.0 × 10^6^ B16F10 cells/mouse. Mice received i.v. injections of ST004 and DTIC formulations at a dose of 3.5 mg/kg and 10 mg/kg, respectively. Treatments were given five consecutive times, once per day. Four experimental groups were established: Control, (that received PBS); Free –ST004; LIP –ST004 (DMPC:DOPE:DSPE-PEG); DTIC (positive control). (**a**) Experimental design, (**b**) Average animal body weight, (**c**) Representative images of lungs with melanoma metastases (black dots), (**d**) Metastasis score established from 1 to 4 according to the number of metastases in the lungs, (**e**) Average metastasis score for each experimental group. Statistical analysis was performed by unpaired t-test with Welch’s correction. * *p* < 0.05 vs. Control. Results are expressed as mean ± SEM (n = 4–5).

After confirming the in vitro antimelanoma activity of the gold complex formulations, the next step was to evaluate the therapeutic potential in subcutaneous (Supplementary Information, Figure S3) and metastatic (Figure 6) murine melanoma models.

In a preliminary subcutaneous model, tumors were induced by an s.c. injection of 1.3 × 10^5^ B16F10 cells/mouse, and treatment began 12 days after (Figure S3a). Mice received i.v. injections of the formulations at a dose of 3.5 mg/kg of body weight, for five consecutive times, once per day. All animals maintained or slightly increased their body weight (Figure S3b). This variation in body weight (<10%) was only observed at the end of the treatment schedule (Figure S4a), which might be associated with the tumor mass increase in all groups. As depicted in Figure S3c, tumor volumes at the end of the experimental protocol were 1260 ± 274, 1103 ± 384 and 1160 ± 286 mm^3^ for Control, Free-ST004 and LIP-ST004 groups, respectively. In previous work accomplished by the present team, an increase in the therapeutic effect for other metal-based complexes, namely copper or iron, following their incorporation in liposomes was achieved in subcutaneous melanoma murine models [18,84]. However, the therapeutic dose used for ST004 was significantly lower (3.5 mg/kg of body weight), and treatment protocol was also shorter.

Based on the absence of therapeutic activity observed in the subcutaneous melanoma model, we also tested ST004 formulations in a B16F10 metastatic model (Figure 6a). The establishment of the metastatic melanoma model was previously optimized by timely monitoring the development of metastases and survival rates [51]. Here, each mouse was inoculated i.v. with 4.0×10^6^ B16F10 cells and treatments began eight days post-induction. A positive control, DTIC, was also included in the animal model (at a dose of 10 mg/kg of body weight), while ST004 formulations were administered at a dose of 3.5 mg/kg once per day for five consecutive days. DTIC dose was selected in accordance with previous literature reports [52,85]. Animal body weight was documented (Figure 6b) and, at the end of the experiment, lungs were macroscopically examined, and a score was attributed according to the number of melanoma metastases (Figure 6c–e). Tissue index of major organs and hepatic biomarkers were assessed as safety parameters (Table S2).

The metastatic B16F10 cancer model most closely simulates the features of advanced melanoma disease in patients, namely high invasiveness, resistance to treatment, and increased mortality. Moreover, as mice are immunocompetent, the complexity of tumor microenvironment and the immune system are maintained, providing a better prediction and understanding of clinical responses [3,51,86,87]. Figure 6b shows a slight decrease (not statistically significant) of body weight by all groups, with LIP-ST004 treatment leading to a partial recovery after the third treatment. In the beginning of the treatment protocol all animals presented an average body weight of (25.8 ± 0.5). At day 13 post-tumor induction, Control, DTIC, Free-ST004 and LIP-ST004 groups displayed an average body weight of 26.1 ± 1.1 g, 22.7 ± 1.0 g, 22.5 ± 1.2 g and 24.4 ± 1.6 g, respectively. In fact, the weight variation (Figure S4b) demonstrated that only animals receiving Lip ST004 were able to recover some body weight at the end of treatment protocol. In the B16F10 metastatic model, the organs that are majorly affected by melanoma metastases are the lungs and brain. Here, the extension of metastases in the lungs was different among tested groups, as confirmed by visual examination (Figure 6c) and respective metastases score (Figure 6d,e). A significant (*p* < 0.05) reduction in the number of metastases was attained in the LIP-ST004 group, with the lowest score of 2.0, followed by Free-ST004 (2.2), DTIC (2.6) and Control (3.0). These findings confirm the efficacy of ST004 formulations, especially when in liposomal form, in limiting the process of melanoma metastization in the lungs.

**Figure 7:**
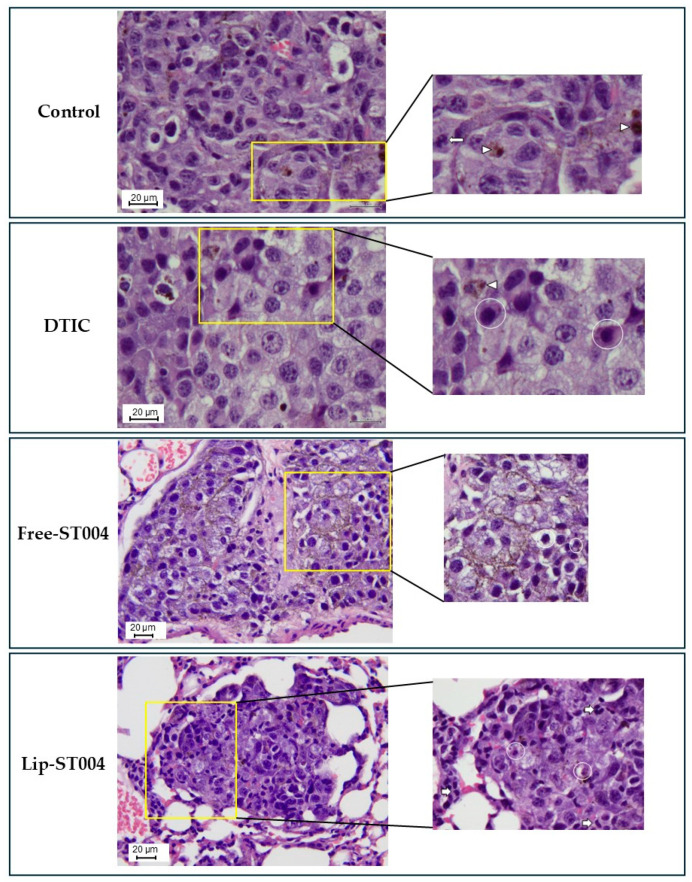
Representative histological images of lung samples stained with H&E for the different groups under study: Control. Neoplastic cells vary from a round to a polygonal profile, with barely evident borders, occasionally containing small amounts of melanic pigment (arrowheads). Long arrows denote a mitotic figure; DTIC. Neoplastic cells maintain typical morphology and occasional melanic pigment (arrowheads). Apoptotic cells are interspersed with viable cells (circles); Free-ST004. Apoptotic cells are interspersed with viable cells (circles); Lip-ST004. Neoplastic cells are intermingled with small infiltrating lymphocytes (short arrows) and apoptotic neoplastic cells (circles). Scale bar in black 20 μm for all the images.

In all animals, histological analysis was also conducted (Figure 7). This evaluation confirmed the presence of multiple foci of varying dimensions of interstitial infiltration by tight nests of round to polygonal neoplastic cells with finely vacuolated cytoplasm, occasionally containing sparse melanin granules; the cells had a large round to ovoid, pale, euchromatic nucleus, with one to four small nucleoli. Furthermore, the group of animals that received Lip-ST004 was associated with overall smaller metastatic nodules when compared to DTIC and Free-ST004. 

The safety of tested formulations in both murine melanoma models was evaluated through tissue index and hepatic enzymes (Table S2). When evaluating new compounds, a well-accepted and sensitive indicator of toxic side effects is organ weight variation [88]. The assessment of tissue index considers the organ weight and the overall body weight of animals, and deviation from normal values may be indicative of organ atrophy and degeneration or hypertrophy, congestion and edema [89,90]. Besides tissue index, the serum levels of AST and ALT are relevant as safety biochemical markers for monitoring potential liver and/or skeletal/cardiac muscle damage [90,91]. These liver enzymes are among those applied in the clinic to identify liver injury. While under normal physiological conditions, the levels of these enzymes may vary within a narrow range, the existence of a pathological condition or liver injury may result in more pronounced oscillations [90,91]. In this work, no major differences among tested groups were detected in terms of tissue index (Tables S1 and S2). In turn, AST and ALT values observed for all groups under study were similar, including the naïve group, being within the reference intervals reported by the mice provider [92].

## 4. Conclusions

In this work, the antimelanoma activity of the gold-based complex ST004, either in free form or after incorporation in long blood circulating liposomes (with a mean size below 100 nm) was assessed in vitro and in vivo.

Results from cytotoxicity assays demonstrated a time- and concentration-dependent antiproliferative activity of ST004 formulations, with LIP-ST004 displaying slightly decreased potency (EC_50_ values 31–95 μM), compared to Free-ST004 (17–58 μM). Unloaded liposomes did not affect cell viability in all tested lipid concentrations. Considering the cell cycle results, the gold-based complex disrupts normal cell proliferation. Free-ST004 halted cell cycle at G0/G1 phase in all murine and human cell lines under study. The LIP-ST004 effect was cell-line-dependent, leading to a G0/G1 halt in B16F10, and to an arrest in S phase in A375 and MNT-1 cells. The organogold compound also revealed a potent inhibitory effect on AQP3 activity, a member of the AQP family closely associated with cancer biological functions and aberrantly expressed in several human cancers. Further studies are necessary to fully explain the observed differences and to elucidate the mechanisms of action of this metallodrug.

The therapeutic evaluation of tested formulations was performed in subcutaneous and metastatic murine melanoma models. In the metastatic B16F10 murine model, a significant reduction in lung metastases was achieved in animals treated with LIP-ST004, compared to Free-ST004 and DTIC. Overall, this study emphasizes the antimelanoma potential of the organometallic gold-based complex and the valuable benefits of using liposomes as a tool to improve its therapeutic index. In future work, the peculiar reactivity of this family of cyclometalated Au(III) compounds, fostering the formation of C–S/Se covalent bonds, can be used to design improved anticancer organogold candidates for covalent drug discovery.

## Figures and Tables

**Table 1:** Physicochemical characterization of LIP-ST004.

Lipid Composition (Molar Ratio)	[ST004]i (μg/mL)	ST004/Lipid(i) (μg/μmol)	ST004/Lipid(f) (μg/μmol)	I.E. (%)	Mean Size (nm) (PdI)	ζ pot. (mV)
DMPC:DOPE:DSPE-PEG (50:45:5)	400	11 ± 3	5 ± 1	46 ± 9	122 ± 2 (<0.1)	−4 ± 2
	1000	29 ± 4	15 ± 5	51 ± 15	104 ± 6 (<0.1)	−5 ± 1

Initial lipid concentration: 30 μmol/mL; initial ST004 concentration: 400 or 1000 μg/mL; DMPC: dimyristoyl phosphatidyl choline (MW = 678); DOPE: dioleoyl phosphatidyl ethanolamine (MW = 744); DSPE-PEG: distearoyl phosphatidyl ethanolamine covalently linked to poly(ethylene glycol) 2000 (MW = 2790); PdI: polydispersity index; ζ pot.: zeta potential; I.E.: incorporation efficiency. Results are expressed as mean ± SD (n = 3 for 400 μg/mL and n = 4 for 1000 μg/mL).

**Table 2:** Half maximal effective concentration (EC_50_) of ST004 formulations and DTIC towards human and murine melanoma cell lines, after 24 and 48 h incubation.

EC_50_ (μM)					
	Free-ST004		LIP-ST004		DTIC
Cell line	24 h	48 h	24 h	48 h	24 h
B16F10	47 ± 3	58 ± 1	87 ± 1	78 ± 3	>100
A375	27 ± 4	17 ± 1	49 ± 2	31 ± 6	>100
MNT-1	37 ± 4	24 ± 5	95 ± 3	55 ± 14	>100

LIP-ST004: DMPC:DOPE:DSPE-PEG(50:45:5); DTIC: dacarbazine; Data are expressed as mean ± SD (n = 2–3).

## Data Availability

Data will be made available on request.

## Supplementary Materials

The following supporting information can be downloaded at: https://www.mdpi.com/article/10.3390/pharmaceutics16121566/s1, Figure S1. Gating strategy for cell cycle analysis by flow cytometry. Debris and doublets were excluded before assessing propidium iodide (PI) fluorescence. A cell cycle histogram was automatically generated for each sample using the MultiCycle AV in FCS Express 7 Software (DeNovo Software); Figure S2. Representative plots of gated B16F10, A375 and MNT-1 melanoma cells in the G0/G1, S, and G2/M phases of cell cycle in the absence (Control) or presence of DTIC at 70 μM (DTIC) or ST004 in free (Free-ST004) or liposomal (LIP-ST004) forms. A cell cycle histogram was automatically generated for each sample using the MultiCycle AV in FCS Express 7 Software (DeNovo Software); Figure S3. Therapeutic effect of tested ST004 formulations in a subcutaneous murine melanoma model. Tumor induction was performed by a s.c. injection of 1.3 × 10^5^ B16F10 cells/mouse. Mice received i.v. injections of the formulations at a dose of 3.5 mg/kg of body weight, five consecutive times, once per day. Three experimental groups were established: Control (that received PBS); Free-ST004; LIP-ST004 (liposomal formulation of ST004, DMPC:DOPE:DSPE-PEG). (a) Experimental design, (b) Average animal weight, and (c) Tumor volume evolution. Tumor volumes were calculated according to the formula: V (mm^3^) = (L × W^2^)/2, where L and W represent the longest and shortest axis of the tumor, respectively. Results are expressed as mean ± SEM (n = 4–5); Figure S4. Weight variation during treatment schedule in a subcutaneous (a) and metastatic (b) murine melanoma model. Mice received i.v. injections of the ST004 formulations at a dose of 3.5 mg/kg of body weight and DTIC at a dose of 10 mg/kg of body weight, five consecutive times, once per day. Experimental groups were established: Control (that received PBS); Free-ST004; LIP-ST004 (liposomal formulation of ST004, DMPC:DOPE:DSPE-PEG) and for the metastatic model DTIC, the positive control. Results are expressed as mean ± SEM (n = 4–5); Table S1. Tissue index and hepatic biomarkers of the subcutaneous murine melanoma model; Table S2. Tissue index and hepatic biomarkers of the metastatic murine melanoma model.
