# Supplementary material for: Liposomal Formulation of an Organogold Complex Enhancing Its Activity as Antimelanoma Agent—In Vitro and In Vivo Studies
Source: Pharmaceutics. 2024 Dec 6;16(12):1566. doi: 10.3390/pharmaceutics16121566 (PMC11678262; doi:10.3390/pharmaceutics16121566)
Supplement: Supplementary file 1 [file pharmaceutics-16-01566-s001.zip › pharmaceutics-3302723-supplementary.pdf]

Supplementary Information

Liposomal formulation of an organogold complex enhancing its activity as antimelanoma agent – in vitro and in vivo studies

Jacinta O. Pinho [1, †], Mariana Coelho [1, †], Catarina Pimpão [1], Jahnobi Konwar [2], Ana Godinho-Santos [1], Rute M. Noiva [3], Sophie R. Thomas [4,5], Angela Casini [4], Graça Soveral [1], M. Manuela Gaspar [1,6,*]

[1] Research Institute for Medicines (iMed.ULisboa) Faculty of Pharmacy, Universidade de Lisboa, 1649-003 Lisboa, Portugal; pinho.jacinta@campus.ul.pt (J.O.P.); mariana.coelho@ff.ulisboa.pt (M.C.); pimpaocatarina@gmail.com (C.P.); agsantos@medicina.ulisboa.pt (A.G.-S.); gsoveral@ff.ulisboa.pt (G.S.)

[2] Faculty of Pharmacy, Jagiellonian University Medical College, 31-008 Krakow, Poland; jahnobi.konwar@student.uj.edu.pl

[3] CIISA, Interdisciplinary Centre of Research in Animal Health, Faculdade de Medicina Veterinária,
Universidade de Lisboa, Av. da Universidade Técnica, 1300-477 Lisboa, Portugal; rute.noiva@gmail.com

[4] Department of Chemistry, School of Natural Sciences, Technical University of Munich, 85747 Garching bei München, Germany; sophie.rebecca.thomas@univie.ac.at (S.R.T.); angela.casini@tum.de (A.C.)

[5] Faculty of Chemistry, Department of Inorganic Chemistry, University of Vienna, Währinger Straße 42, A-1090 Wien, Austria

[6] IBEB —-Institute of Biophysics and Biomedical Engineering, Faculty of Sciences, Universidade de Lisboa, 1749-016 Lisboa, Portugal

* Correspondence: mgaspar@ff.ulisboa.pt

[†] These authors contributed to this work equally.

Supplementary Results

A) Cell cycle analysis

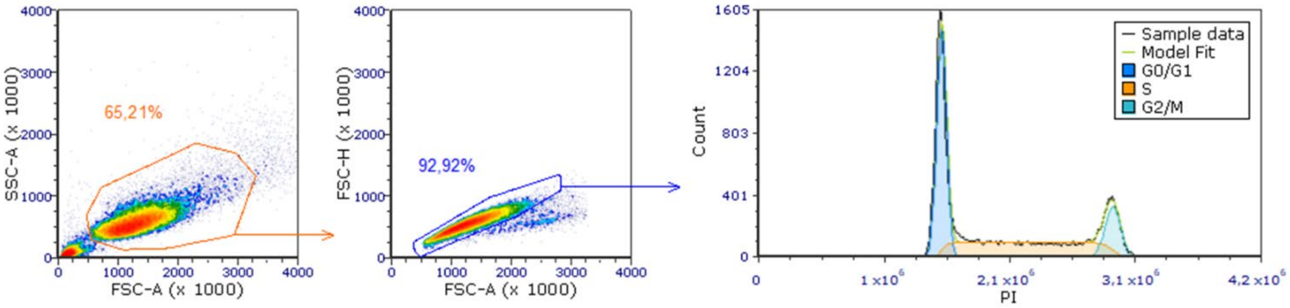

Figure S1. Gating strategy for cell cycle analysis by flow cytometry. Debris and doublets were excluded before assessing propidium iodide (PI) fluorescence. A cell cycle histogram was automatically generated for each sample using the MultiCycle AV in FCS Express 7 Software (DeNovo Software).

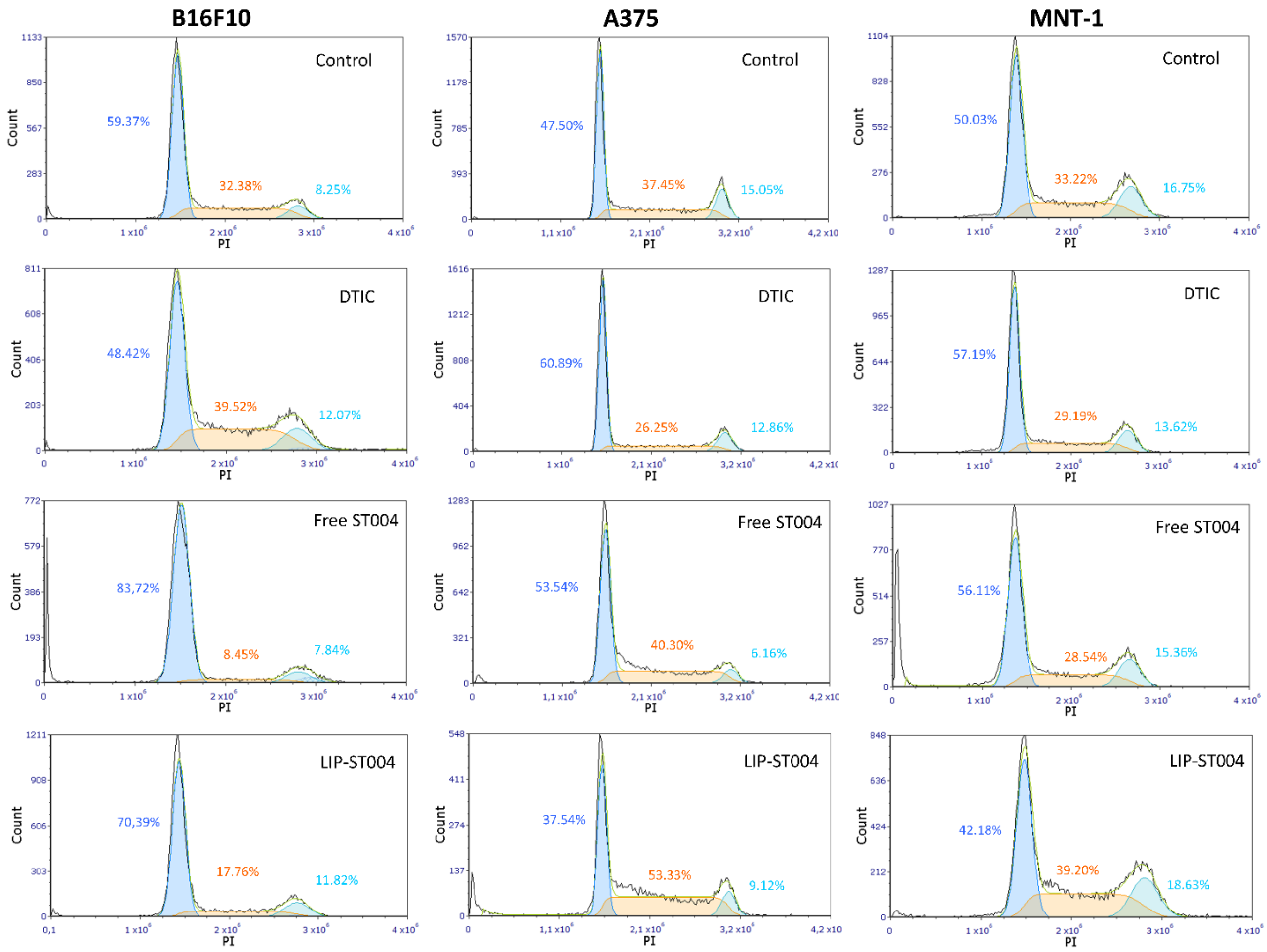

Figure S2. Representative plots of gated B16F10, A375 and MNT-1 melanoma cells in the G0/G1, S, and G2/M phases of cell cycle in the absence (Control) or presence of DTIC at 70 µM (DTIC) or ST004 in free (Free-ST004) or liposomal (LIP-ST004) forms. A cell cycle histogram was automatically generated for each sample using the MultiCycle AV in FCS Express 7 Software (DeNovo Software).

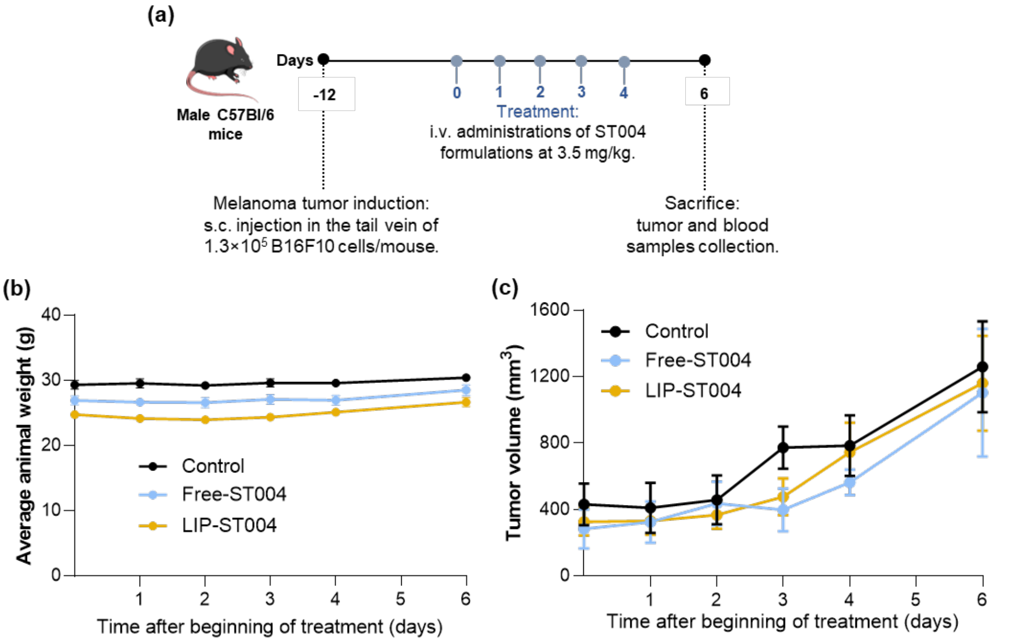

Figure S3. Therapeutic effect of tested ST004 formulations in a subcutaneous murine melanoma model. Tumor induction was performed by a s.c. injection of 1.3×10^5 B16F10 cells/mouse. Mice received i.v. injections of the formulations at a dose of 3.5 mg/kg of body weight, five consecutive times, once per day. Three experimental groups were established: Control (that received PBS); Free-ST004; LIP-ST004 (liposomal formulation of ST004, DMPC:DOPE:DSPE-PEG). **(a)** Experimental design, **(b)** Average animal weight, and **(c)** Tumor volume evolution. Tumor volumes were calculated according to the formula: V (mm^3) = ($L \times W^2$)/2, where L and W represent the longest and shortest axis of the tumor, respectively. Results are expressed as mean ± SEM (n = 4-5).

Table S1. Tissue index and hepatic biomarkers of the subcutaneous murine melanoma model.

	Tissue Index (AVG ± SEM)				Hepatic Biomarkers (AVG ± SEM)	
	**Liver**	**Spleen**	**Lungs**	**Kidneys**	**AST (U/L)**	**ALT (U/L)**
**Naïve**	22.1 ± 0.6	5.1 ± 0.1	6.3 ± 0.6	10.7 ± 0.5	19.2 ± 3.5	6.1 ± 2.6
**Control**	21.3 ± 1.3	5.1 ± 0.2	6.5 ± 0.3	10.7 ± 0.2	9.6 ± 4.0	5.7 ± 2.3
**DTIC**	24.4 ± 0.4	5.4 ± 0.1	7.2 ± 0.2	11.2 ± 0.1	26.8 ± 4.6	4.1 ± 2.2
**Free-ST004**	23.4 ± 0.3	5.3 ± 0.2	7.4 ± 0.1	10.4 ± 0.1	24.8 ± 5.3	6.6 ± 3.6
**LIP-ST004**	23.6 ± 0.5	6.4 ± 0.3	7.8 ± 0.3	10.7 ± 0.1	21.5 ± 10.8	7.4 ± 1.9

Table S2. Tissue index and hepatic biomarkers of the metastatic murine melanoma model.

	**Tissue Index (AVG ± SEM)**					**Hepatic Biomarkers (AVG ± SEM)**	
	**Liver**	**Spleen**	**Lungs**	**Kidneys**	**Brain**	**AST (U/L)**	**ALT (U/L)**
**Control**	20.6 ± 0.3	5.5 ± 0.2	7.7 ± 0.2	10.9 ± 0.1	12.6 ± 0.4	12.2 ± 1.6	9.6 ± 2.5
**DTIC**	20.9 ± 0.4	5.5 ± 0.1	8.1 ± 0.1	11.0 ± 0.1	12.1 ± 0.4	4.8 ± 0.7	6.1 ± 0.4
**Free-ST004**	18.2 ± 0.5	6.0 ± 0.4	8.0 ± 0.4	11.6 ± 0.4	11.7 ± 0.7	12.2 ± 5.0	3.5 ± 0.7
**LIP-ST004**	20.8 ± 0.5	6.4 ± 0.2	7.4 ± 0.3	10.6 ± 0.3	11.9 ± 0.6	21.4 ± 8.8	5.2 ± 1.6

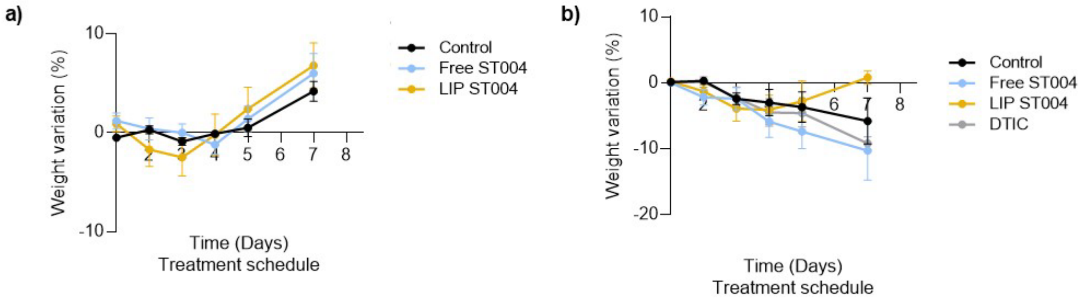

Figure S4. Weight variation during treatment schedule in a subcutaneous (a) and metastatic (b) murine melanoma model. Mice received i.v. injections of the ST004 formulations at a dose of 3.5 mg/kg of body weight and DTIC at a dose of 10 mg/kg of body weight, five consecutive times, once per day. Experimental groups were established: Control (that received PBS); Free-ST004; LIP-ST004 (liposomal formulation of ST004, DMPC:DOPE:DSPE-PEG) and for the metastatic model DTIC, the positive control. Results are expressed as mean ± SEM (n = 4-5).

The weight variation in percentage during treatment schedule (Figure S4a and S4b) was determined according to the following equation:

$$weight\ variation\ (\%) = \frac{Weight_x - Weight_y}{Weight_y} \times 100$$

where $Weight_x$ corresponds to the weight of the animal on each day of treatment and $Weight_y$ corresponds to the weight of the respective animal on the day before the beginning of treatment.
